# Preclinical Evaluation of Recombinant Microbial Glycoside Hydrolases in the Prevention of Experimental Invasive Aspergillosis

**DOI:** 10.1128/mBio.02446-21

**Published:** 2021-09-28

**Authors:** Hanna Ostapska, Deepa Raju, Melanie Lehoux, Ira Lacdao, Stephanie Gilbert, Piyanka Sivarajah, Natalie C. Bamford, Perrin Baker, Thi Tuyet Mai Nguyen, Caitlin A. Zacharias, Fabrice N. Gravelat, P. Lynne Howell, Donald C. Sheppard

**Affiliations:** a Department of Microbiology and Immunology, McGill University, Montreal, Canada; b Infectious Disease and Immunity in Global Health Program, McGill University Health Centre, Montreal, Canada; c McGill Interdisciplinary Initiative in Infection and Immunity, Montreal, Canada; d Program in Molecular Medicine, Research Institute, The Hospital for Sick Children, Toronto, Canada; e Department of Biochemistry, University of Toronto, Toronto, Canada; f Department of Medicine, McGill University, Montreal, Canada; Vallabhbhai Patel Chest Institute

**Keywords:** *Aspergillus fumigatus*, antifungal agents, antimicrobial combinations, biofilm, exopolysaccharide, filamentous fungi, galactosaminogalactan (GAG), glycoside hydrolase (GH), pulmonary aspergillosis

## Abstract

Aspergillus fumigatus is a ubiquitous mold that can cause invasive pulmonary infections in immunocompromised patients. Within the lung, A. fumigatus forms biofilms that can enhance resistance to antifungals and immune defenses. Aspergillus biofilm formation requires the production of a cationic matrix exopolysaccharide, galactosaminogalactan (GAG). In this study, recombinant glycoside hydrolases (GH)s that degrade GAG were evaluated as antifungal agents in a mouse model of invasive aspergillosis. Intratracheal GH administration was well tolerated by mice. Pharmacokinetic analysis revealed that although GHs have short half-lives, GH prophylaxis resulted in reduced fungal burden in leukopenic mice and improved survival in neutropenic mice, possibly through augmenting pulmonary neutrophil recruitment. Combining GH prophylaxis with posaconazole treatment resulted in a greater reduction in fungal burden than either agent alone. This study lays the foundation for further exploration of GH therapy in invasive fungal infections.

## INTRODUCTION

The filamentous mold Aspergillus fumigatus is the most common cause of invasive pulmonary aspergillosis (1, 2), a necrotizing pneumonia that can develop in immunocompromised patients, such as those receiving chemotherapy or undergoing hematopoietic stem cell transplantation (2, 3). Despite currently available antifungal therapy, A. fumigatus infection-related mortality in these patients remains high (1). There is therefore an urgent need for the development of new preventative and treatment strategies for invasive aspergillosis. One approach toward improving patient outcome is to target molecules involved in fungal pathogenesis.

During pulmonary infection, A. fumigatus forms biofilms that help protect the fungus from host immune defenses and antifungal agents (4). Production of the exopolysaccharide galactosaminogalactan (GAG) enhances A. fumigatus biofilm formation *in vitro* (5–7). GAG is a cationic heteropolysaccharide composed of α-1,4-linked d-galactose and partially deacetylated *N*-acetyl-d-galactosamine (GalNAc) (8). The partial deacetylation of GalNAc residues following GAG synthesis renders the polymer cationic and adhesive to anionic surfaces, such as glass, plastic, and human cells (5–7). GAG is secreted during vegetative growth and is found both bound to hyphae and within the matrix of fungal biofilms (4, 5, 8). Hyphal-associated GAG plays a number of roles in the pathogenesis of fungal infection, including concealing surface-exposed fungal pathogen-associated molecular patterns such as β-glucan from immune detection by pattern recognition receptors, enhancing resistance to killing by neutrophil extracellular traps and mediating adherence of hyphae to substrates, including fibronectin and epithelial cells (5, 9). Secreted GAG has been reported to induce neutrophil apoptosis and increase the production of immunosuppressive interleukin-1 (IL-1) receptor antagonist by macrophages and neutrophils, thus compromising both the innate and adaptive immune response (8, 10). Consistent with the multiple roles of GAG in virulence, GAG-deficient strains exhibit reduced virulence in mouse models of invasive infection, and overexpression of GAG in the nonpathogenic Aspergillus nidulans increased virulence of this organism in immunosuppressed mice (5, 6, 9).

Given the importance of GAG in the pathogenesis of invasive pulmonary aspergillosis, we hypothesized that targeting GAG is an effective strategy to attenuate A. fumigatus virulence. GAG synthesis is dependent on a cluster of five genes that are predicted to encode carbohydrate active enzymes. Two of these genes, *ega3* and *sph3*, encode glycoside hydrolase (GH) enzymes that cleave GAG (6, 7, 11, 12). Structure-function studies using soluble recombinant GH domains from these proteins (Sph3_h_ and Ega3_h_) revealed that Sph3_h_ is an endo-α-1,4-*N*-acetyl-d-galactosaminidase that cleaves GalNAc-GalNAc linkages within acetylated regions of GAG, whereas Ega3_h_ is an endo-α-1,4-d-galactosaminidase with specificity for deacetylated regions of the polymer (12, 13). Treatment with soluble recombinant GH domains of Sph3_h_ and Ega3_h_ can hydrolyze GAG and disrupt A. fumigatus biofilms *in vitro* (11, 12, 14).

The biofilm-forming bacterium Pseudomonas aeruginosa secretes Pel, a cationic exopolysaccharide that is believed to be composed predominantly of GalNAc with small amounts of *N*-acetylated d-glucosamine, one or both of which are partially deacetylated (15, 16). The Pel biosynthetic machinery includes a multidomain enzyme, PelA, containing a GH domain, PelA_h_ (15). Like Sph3_h_, the soluble recombinant GH domain PelA_h_ is an endo-α-1,4-*N*-acetyl-d-galactosaminidase that can cleave GAG; however, unlike Sph3_h_, PelA_h_ can also cleave GalNAc-GalNAc linkages within partially deacetylated regions of the GAG polymer (13). Thus, in addition to degrading Pel-dependent P. aeruginosa biofilms, the recombinant GH domain of PelA_h_ can also disrupt A. fumigatus biofilms (14, 17). Interestingly, Ega3_h_ also exhibits cross-kingdom activity and can disrupt Pel-dependent P. aeruginosa biofilms, suggesting that GAG and Pel contain regions of similar composition (12). Treatment with soluble recombinant Sph3_h_ and PelA_h_ domains enhances the activity of antifungals posaconazole, amphotericin B, and caspofungin against A. fumigatus *in vitro* (14). Fluorometric studies further demonstrated enhanced intracellular penetration of posaconazole in Sph3_h_-treated hyphae, suggesting a role for GH therapy in combination with current antifungals (14).

Here, the tolerability and anti-Aspergillus activity of recombinant GH therapy with Sph3_h_, PelA_h_, and Ega3_h_ was evaluated *in vivo* using immunocompromised mouse models of invasive aspergillosis (6, 18). Single-dose intratracheal Sph3_h_, PelA_h_, and Ega3_h_ administration was well tolerated by uninfected mice. Prophylaxis with a single dose of GH at the time of infection attenuated A. fumigatus virulence in two immunocompromised mouse models of invasive pulmonary aspergillosis. In addition, prophylaxis with Sph3_h_ in combination with posaconazole treatment enhanced the antifungal activity of posaconazole against A. fumigatus in a neutropenic mouse model of invasive pulmonary aspergillosis. These results suggest that GH therapy is a promising approach for the prevention of invasive aspergillosis.

## RESULTS

### Intratracheal GH treatment is well tolerated by mice.

Previously, structure-function studies of Sph3_h_ and PelA_h_ produced in Escherichia coli and Ega3_h_ produced in Pichia pastoris (Ega3_h_-*Pp*) demonstrated that these soluble recombinant GH domains can disrupt A. fumigatus biofilms by degrading GAG (11–13). In addition, we found that intratracheal administration of a single dose of up to 500 μg Sph3_h_ was generally well tolerated by mice (14). To extend these findings, the tolerability of intratracheal Sph3_h_, PelA_h_, and Ega3_h_-*Pp* was examined in greater detail. Immunocompetent BALB/c mice were administered up to 500 μg of intratracheal Sph3_h_, PelA_h_, or Ega3_h_-*Pp* and monitored for changes in weight and temperature and then euthanized 7 days later for measures of pulmonary injury and inflammation. Treatment with a single dose of up to 500 μg of Sph3_h_, PelA_h_, or Ega3_h_-*Pp* was well tolerated by mice, without signs of respiratory distress or mortality. GH-treated mice exhibited no difference in body weight and temperature compared with mice treated with buffer alone (see Fig. S1 and S2 in the supplemental material).

10.1128/mBio.02446-21.1 FIG S1 Intratracheal GH therapy is well tolerated by mice. Body weight of immunocompetent BALB/c mice following intratracheal treatment with the indicated single doses of Sph3_h_ (A), PelA_h_ (B), or Ega3_h_-*Pp* (C). Each point represents the body weight of ≥5 mice per group. ns indicates no significant difference in the change in body weight of mice treated with 500 μg GH relative to buffer-treated mice on the same day, as determined by two-way ANOVA with Dunnett’s multiple-comparison test. Download FIG S1, PDF file, 0.1 MB. Copyright © 2021 Ostapska et al. 2021 Ostapska et al. https://creativecommons.org/licenses/by/4.0/ This content is distributed under the terms of the Creative Commons Attribution 4.0 International license.

10.1128/mBio.02446-21.2 FIG S2 Intratracheal GH therapy is well tolerated by mice. Body temperature of immunocompetent BALB/c mice following intratracheal treatment with the indicated single doses of Sph3_h_ (A), PelA_h_ (B), or Ega3_h_-*Pp* (C). Points represent the body temperature of ≥5 mice per group. ns indicates no significant difference in the change in body temperature of mice treated with 500 μg GH relative to buffer-treated mice as determined by two-way ANOVA with Dunnett’s multiple-comparison test. Download FIG S2, PDF file, 0.1 MB. Copyright © 2021 Ostapska et al. 2021 Ostapska et al. https://creativecommons.org/licenses/by/4.0/ This content is distributed under the terms of the Creative Commons Attribution 4.0 International license.

**FIG 1:**
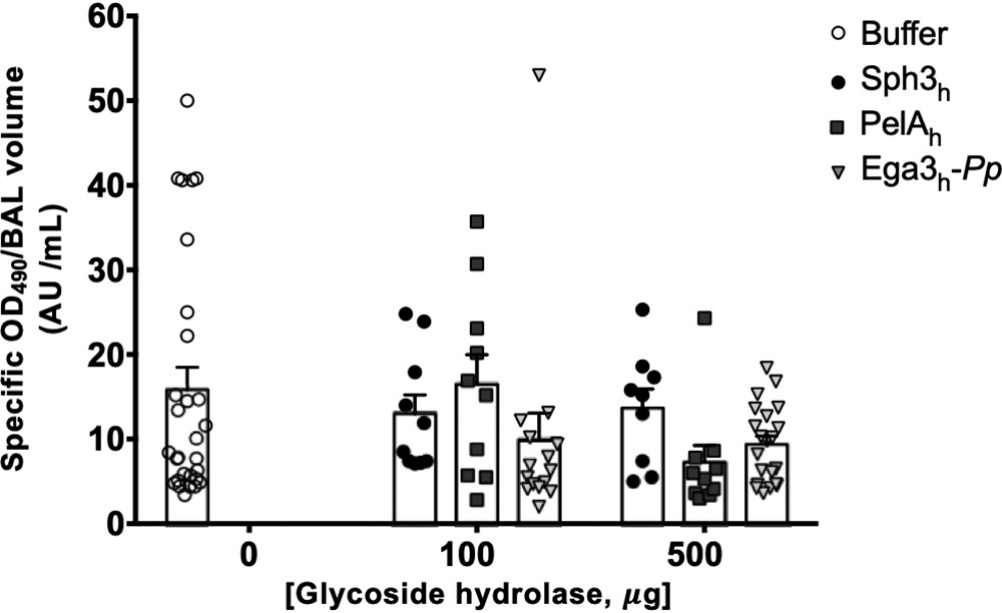
Intratracheal GH therapy does not induce pulmonary damage. Lactate dehydrogenase activity was quantified in bronchoalveolar lavage (BAL) fluid from mouse lungs of immunocompetent BALB/c mice 7 days after intratracheal administration of a single dose of 100 or 500 μg of Sph3_h_, PelA_h_, or Ega3_h_-*Pp*. Bars represent the means ± standard errors from at least 2 independent experiments with ≥9 mice per group. No significant differences were found between any test condition and the buffer-treated group (*P > *0.05) as determined by two-way ANOVA with Dunnett’s multiple-comparison test.

To test if intratracheal GH therapy induced pulmonary injury, pulmonary damage was assessed by measuring lactate dehydrogenase activity in bronchoalveolar lavage fluid from mouse lungs. No significant increase in lactate dehydrogenase activity was detected in the bronchoalveolar lavage fluid from mice treated with 500 μg Sph3_h_, PelA_h_, or Ega3_h_-*Pp* compared with mice treated with buffer alone (Fig. 1) (14), suggesting that single-dose GH treatment does not induce pulmonary injury in mice. Consistent with these findings, histological examination of pulmonary sections did not reveal any differences between GH-treated and buffer-treated mice (Fig. S3).

10.1128/mBio.02446-21.3 FIG S3 Intratracheal GH therapy is well tolerated by mice. Hematoxylin- and eosin-stained sections of lungs obtained from immunocompetent BALB/c mice 7 days after intratracheal treatment with a single dose of 500 μg of Sph3_h_ (A), PelA_h_ (B), Ega3_h_-*Pp* (C), or PBS (D). Representative images from 3 mice imaged at 40× (scale bar, 50 μm). Download FIG S3, PDF file, 1.6 MB. Copyright © 2021 Ostapska et al. 2021 Ostapska et al. https://creativecommons.org/licenses/by/4.0/ This content is distributed under the terms of the Creative Commons Attribution 4.0 International license.

**FIG 2:**
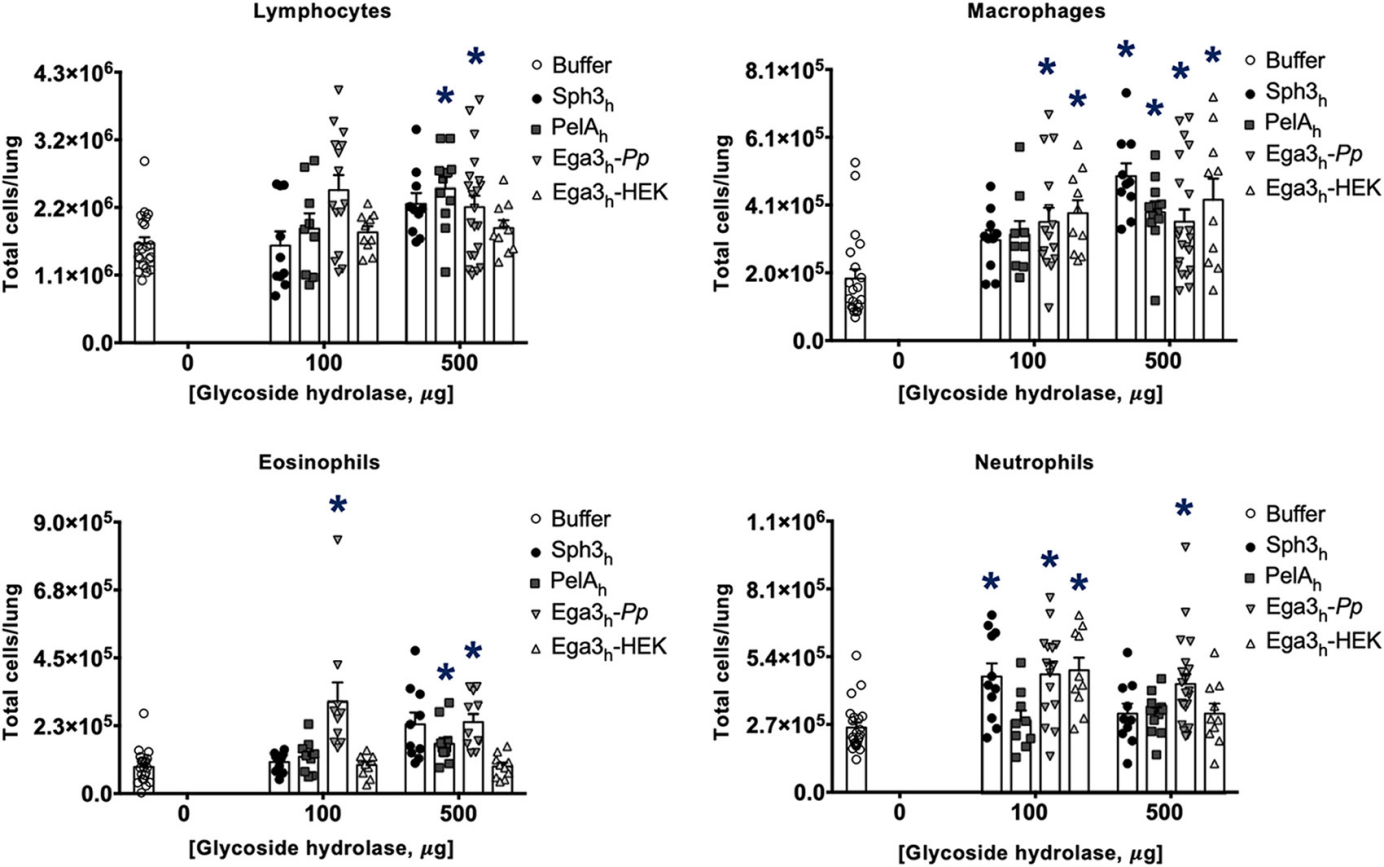
Pulmonary leukocyte numbers following intratracheal GH therapy. Immunocompetent BALB/c mice were treated intratracheally with a single dose of 100 or 500 μg of Sph3_h_, PelA_h_, Ega3_h_*-Pp*, or Ega3_h_-HEK. Pulmonary leukocyte populations, including lymphocytes, macrophages, eosinophils, and neutrophils, were quantified by flow cytometry 7 days after GH treatment. Bars represent the means ± standard errors from 2 independent experiments with ≥9 mice per group. A significant difference is indicated (*, *P < *0.05) relative to the buffer-treated group as determined by two-way ANOVA with Dunnett’s multiple-comparison test.

To further probe the host response to intratracheal GH treatment, pulmonary leukocytes from GH-treated mice were quantified by flow cytometry (Fig. 2). There was no significant difference in pulmonary lymphocyte numbers between mice treated with either the 100- or 500-μg dose of Sph3_h_ and mice treated with buffer alone. Sph3_h_ treatment also had no effect on macrophage or eosinophil numbers at the 100-μg dose, while a small but significant increase in the number of these cells was detected in mice treated with 500 μg Sph3_h_. Although a significant increase in pulmonary neutrophils was observed following treatment with 100 μg Sph3_h_, this was not observed in mice treated with the higher 500-μg dose of Sph3_h_. In the case of PelA_h_ treatment, increases in pulmonary lymphocytes and macrophages were observed at the higher GH dose. In contrast to Sph3_h_ or PelA_h_, treatment with all doses of Ega3_h_-*Pp* was associated with a significant increase in leukocyte populations, including lymphocytes, macrophages, eosinophils, and neutrophils. Taken together, these data suggest that while 500 μg of Sph3_h_ or PelA_h_ is near the maximal tolerated intratracheal dose, Ega3_h_-*Pp* is significantly more inflammatory and may be less tolerated by mice.

Ega3_h_-*Pp* was produced in yeast, unlike Sph3_h_ and PelA_h_, which were produced in bacteria. As eukaryotic proteins differ from bacterial proteins in their glycosylation patterns (19) and contaminating fungal β-glucan could be copurified with Ega3_h_-*Pp* (20), we hypothesized that the inflammatory response to Ega3_h_-*Pp* treatment is a consequence of fungal glycosylation patterns or trace amounts of β-glucan. We therefore turned to the production of the soluble domain of Ega3_h_ in the human embryonic cell line HEK293-S (Ega3_h_-HEK). In contrast to treatment with yeast-derived Ega3_h_-*Pp*, no significant increase in pulmonary lymphocyte and eosinophil numbers was observed in mice treated with either 100 or 500 μg Ega3_h_-HEK. Although a significant increase in pulmonary neutrophil numbers was detected following treatment with 100 μg Ega3_h_-HEK, this was not seen in mice treated with 500 μg Ega3_h_-HEK. As with Ega3_h_-*Pp*, a significant increase in pulmonary macrophage numbers was detected at both 100 and 500 μg Ega3_h_-HEK. These data suggest that production of Ega3_h_ in a mammalian cell line induced a lower inflammatory response than production in yeast.

### GHs exhibited short pulmonary half-lives.

**FIG 3:**
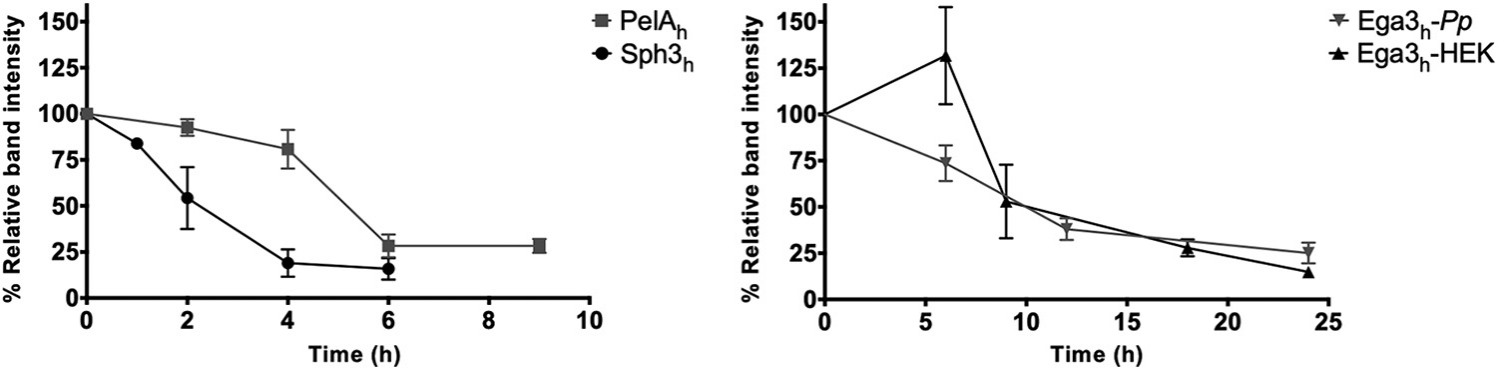
Pulmonary GH pharmacokinetic profile. Leukopenic mice were treated intratracheally with a single dose of 500 μg of Sph3_h_, PelA_h_, Ega3_h_-*Pp*, or Ega3_h_-HEK and then sacrificed at the indicated time points. Lung homogenates were assessed by Western blotting. Dots represent the means ± standard errors from band intensities normalized to total band intensity at 0 h from at least 1 independent experiment with ≥5 mice per time point.

To inform the design of further efficacy studies, the pharmacokinetics of the GHs were determined in leukopenic mice. Cyclophosphamide- and cortisone acetate-treated mice were given a single intratracheal dose of 500 μg Sph3_h_, PelA_h_, Ega3_h_-*Pp*, or Ega3_h_-HEK. At select time points the mice were euthanized and their lungs were harvested and homogenized in a cocktail of protease inhibitors to prevent degradation of GHs. Lung homogenates were assessed by Western blotting using rabbit anti-GH antibodies, and the half-lives of the GHs in the lungs were determined by densitometry. Ega3_h_-*Pp* and Ega3_h_-HEK displayed longer half-lives of approximately 9 h compared to those of PelA_h_ or Sph3_h_, with half-lives of approximately 5 and 3 h, respectively (Fig. 3). These data suggest that Ega3_h_-*Pp* and Ega3_h_-HEK are more stable in the lungs than either PelA_h_ or Sph3_h_. Given that Ega3_h_-HEK had a similar half-life but induced a lower inflammatory response than Ega3_h_-*Pp*, Ega3_h_-HEK was used in all subsequent *in vivo* experiments.

### Pulmonary GH prophylaxis attenuates fungal virulence in an immunocompromised mouse model of invasive pulmonary aspergillosis.

**FIG 4:**
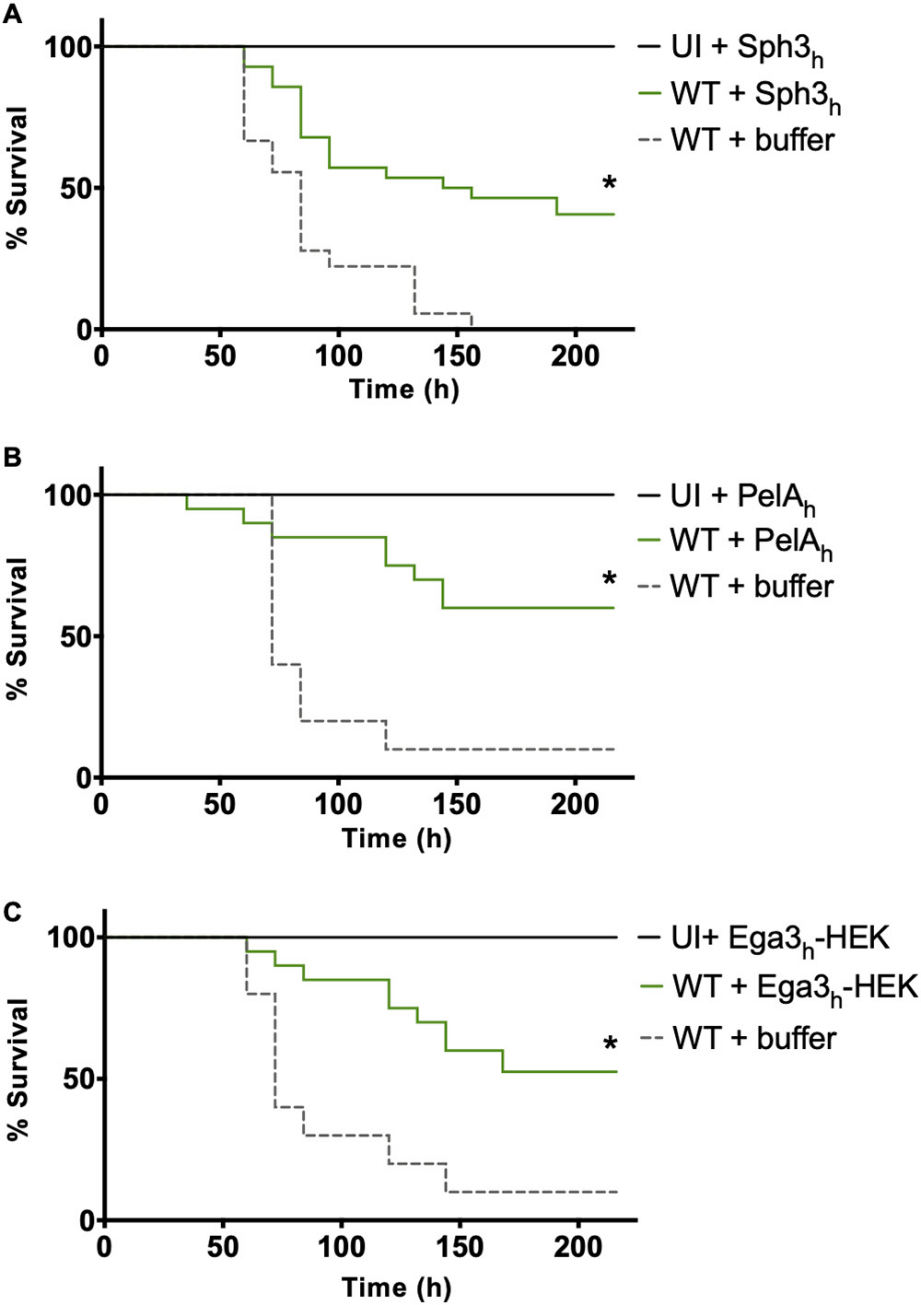
Single-dose intratracheal GH prophylaxis attenuates fungal virulence in a neutropenic mouse model of pulmonary invasive aspergillosis. Survival of neutropenic mice that were intratracheally infected with 5 × 10^6^ wild-type (WT) A. fumigatus conidia and coadministered with a single dose of 500 μg of Sph3_h_ (A), PelA_h_ (B), or Ega3_h_-HEK (C). Kaplan-Meier curves represent 3 independent experiments for Sph3_h_ and 2 independent experiments for PelA_h_ and Ega3_h_-HEK and with ≥10 mice per group. A significant difference is indicated (*, *P < *0.01) relative to the buffer-treated WT group as determined by Wilcoxon-rank test.

To test the antifungal activity of GH prophylaxis *in vivo*, the effects of a single GH dose on survival of A. fumigatus-infected mice were assessed in a neutropenic model of invasive aspergillosis. Mice were rendered neutropenic with anti-Ly6G antibody treatment and then infected with A. fumigatus with or without the coadministration of 500 μg of Sph3_h_, PelA_h_, or Ega3_h_-HEK (Fig. S4A). GH prophylaxis was well tolerated in infected mice, and there was no clinical evidence of central nervous system dissemination (head tilt or leg drag) in GH- or buffer-treated mice. GH prophylaxis with a single dose of Sph3_h_, PelA_h_, or Ega3_h_-HEK resulted in a significant increase in survival of infected mice compared to untreated infected mice (Fig. 4).

10.1128/mBio.02446-21.4 FIG S4 Experimental design of GH evaluation in immunocompromised mouse models of invasive aspergillosis. (A) Neutrophil depletion model. Mice were rendered neutropenic by intraperitoneal injection of 200 μg anti-Ly6G antibody starting 24 h prior to infection and every 48 h thereafter. Mice were then intratracheally infected with 5 × 10^6^ A. fumigatus conidia with or without GHs, and survival was monitored. (B) Leukopenia model. Mice were rendered leukopenic by subcutaneous injection with 250 mg of cortisone acetate per kg of body weight 48 h prior to infection and at 72 h after infection and by intraperitoneal injection with 250 mg cyclophosphamide per kg 48 h prior to infection and 200 mg at 72 h after infection. Mice were intratracheally infected with 5 × 10^3^ A. fumigatus conidia with and without GHs, and pulmonary fungal burden was measured at 96 h. (C) Sph3_h_-posaconazole combination studies. Mice were rendered neutropenic as for panel A. Mice were intratracheally infected with 5 × 10^3^ A. fumigatus conidia coadministered with or without a single dose of 500 μg Sph3_h_ and then treated by oral gavage with 2.5 mg/kg posaconazole every 12 h, and pulmonary fungal burden was measured 48 h after infection. Download FIG S4, PDF file, 0.03 MB. Copyright © 2021 Ostapska et al. 2021 Ostapska et al. https://creativecommons.org/licenses/by/4.0/ This content is distributed under the terms of the Creative Commons Attribution 4.0 International license.

**FIG 5:**
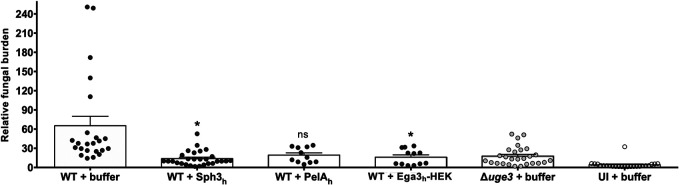
Single-dose pulmonary GH prophylaxis attenuates fungal virulence in a leukopenic mouse model of pulmonary invasive aspergillosis. Pulmonary fungal burden determined by pulmonary galactomannan quantification after 4 days of infection with 5 × 10^3^ conidia of wild-type (WT) or Δ*uge3* strains of A. fumigatus coadministered with a single dose of 500 μg of Sph3_h_, PelA_h_, or Ega3_h_-HEK. Bars represent at least 2 independent experiments with ≥11 mice per group. A significant difference is indicated (*, *P < *0.05), and no significant difference is indicated by ns (*P = *0.055) relative to the wild-type-infected buffer-treated (WT + buffer) group, as determined by Kruskal-Wallis test with Dunn’s multiple-comparison test. UI, uninfected mice.

To confirm and extend these findings, the effects of GH prophylaxis on A. fumigatus virulence were assessed in a second mouse model of invasive pulmonary aspergillosis. Mice were rendered leukopenic with cyclophosphamide and cortisone acetate treatment and then intratracheally infected with A. fumigatus conidia and coadministered a single dose of 500 μg of Sph3_h,_ PelA_h_, or Ega3_h_-HEK (Fig. S4B). Four days following infection, pulmonary galactomannan content was determined as a measure of fungal burden (5, 14, 21). A. fumigatus-infected mice treated with Sph3_h_ or Ega3_h_-HEK were found to have a significantly lower fungal burden than buffer-treated, infected mice (Fig. 5). Indeed, the pulmonary fungal burden of Sph3_h_- or Ega3_h_-HEK-treated mice was not statistically different from that observed in mice infected with the GAG-deficient Δ*uge3* A. fumigatus strain (Fig. 5) (14). A similar trend in the reduction of pulmonary fungal burden was seen in mice receiving PelA_h_, although this difference failed to reach statistical significance (Fig. 5). Collectively the results of our studies in these two models suggest that GH administration can protect against invasive aspergillosis in mice.

**FIG 6:**
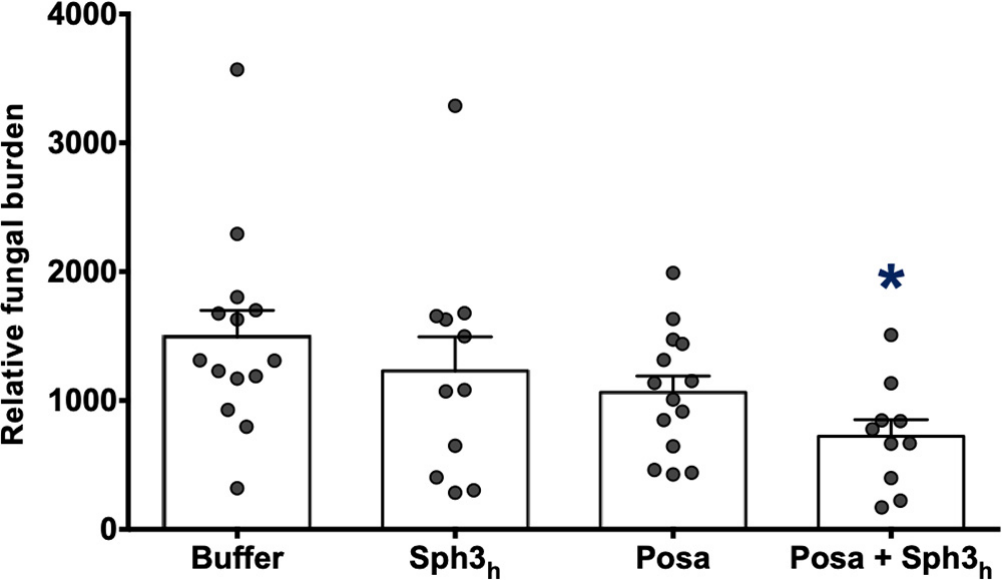
Sph3_h_ prophylaxis enhances posaconazole activity in a neutropenic mouse model of pulmonary invasive aspergillosis. Neutropenic mice were intratracheally infected with 5 × 10^3^ wild-type A. fumigatus conidia coadministered with or without 500 μg Sph3_h_ and then treated as indicated with 2.5 mg/kg posaconazole every 12 h for 2 days. Pulmonary fungal burden was determined by pulmonary galactomannan quantification. Bars represent 3 independent experiments with ≥17 mice per group. A significant difference is indicated (*, *P = *0.0063) relative to all groups as determined by Kruskal-Wallis test with Dunn’s multiple-comparison test. Posa, posaconazole.

GH prophylaxis enhances the susceptibility of A. fumigatus to the antifungal posaconazole *in vitro* (14). To determine if GH prophylaxis can enhance the antifungal activity of posaconazole *in vivo*, the combination of Sph3_h_ with the antifungal posaconazole was compared to monotherapy with either agent alone in the neutropenic model of invasive aspergillosis (Fig. S4C). Neutropenic mice were intratracheally infected with A. fumigatus conidia and administered a single dose of 500 μg Sph3_h_, a submaximal dose of posaconazole (2.5 mg/kg every 12 h), or a combination of the two. Two days following infection, pulmonary galactomannan content was determined as a measure of fungal burden (5, 14, 21). At this time point, prophylaxis with Sph3_h_ or treatment with posaconazole alone resulted in a trend toward reduced fungal burden compared with buffer-treated mice. In contrast, a significant reduction in fungal burden was observed in infected mice receiving Sph3_h_-posaconazole in combination (Fig. 6). These findings suggest that, as was seen *in vitro*, Sph3_h_ prophylaxis enhances the antifungal activity of posaconazole and that GH-antifungal therapy is more effective than either therapy alone.

### The catalytic activity of Sph3_h_ prophylaxis is dispensable for antifungal activity.

**FIG 7:**
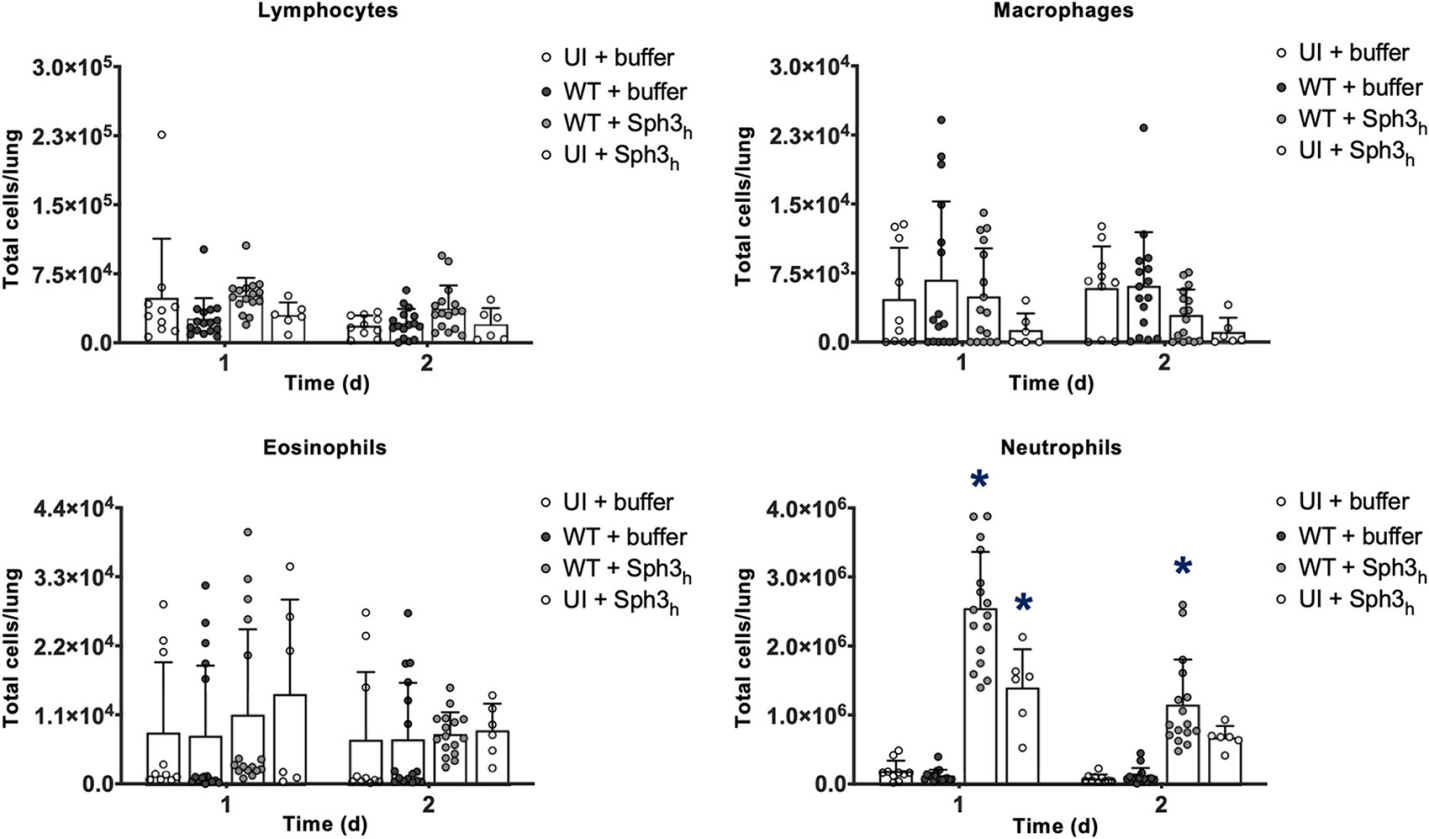
Single-dose intratracheal Sph3_h_ prophylaxis is associated with early pulmonary neutrophil recruitment. Leukopenic mice were intratracheally infected with 5 × 10^3^ wild-type (WT) A. fumigatus conidia coadministered with or without 500 μg Sph3_h_. Pulmonary leukocyte populations were quantified by flow cytometry at 1 day and 2 days after Sph3_h_ prophylaxis. Bars represent the means ± standard errors from 3 independent experiments for uninfected buffer-treated (UI + buffer), wild-type-infected buffer-treated (WT + buffer), and wild-type-infected Sph3_h_-treated (WT + Sph3_h_), and 2 independent experiments for uninfected Sph3_h_-treated (UI + Sph3_h_) group, with ≥6 mice per group. A significant increase in leukocyte populations is indicated (*, *P < *0.0001) relative to the uninfected buffer-treated (UI + buffer) group at 1 day and 2 days as determined by two-way ANOVA with Tukey’s multiple-comparison test.

GAG is absent from resting and swollen spores of A. fumigatus and is only produced following germination and during hyphal growth. Given the short pulmonary half-lives of GHs, it is possible that these enzymes augment host resistance through activating host inflammatory responses rather than via GAG degradation. To investigate this hypothesis, the early immune response to GH therapy was probed by pulmonary leukocyte quantification in Sph3_h_-treated mice during early A. fumigatus infection (Fig. 7). Leukopenic mice were intratracheally infected with A. fumigatus conidia coadministered with a single dose of 500 μg Sph3_h_ (Fig. S4B), and the lungs were harvested after 24 and 48 h for quantification of pulmonary leukocyte populations. A significant increase in neutrophil numbers was detected in both uninfected and A. fumigatus-infected Sph3_h_-treated mice at both 1 and 2 days after Sph3_h_ administration. A trend toward increased eosinophil populations was observed in uninfected Sph3_h_-treated mice on day one; however, this failed to reach significance. No significant increase in pulmonary lymphocyte or macrophage numbers was observed. Together, these observations suggest that Sph3_h_-driven recruitment of pulmonary neutrophils contributes to the activity of this agent in the prevention of invasive aspergillosis.

**FIG 8:**
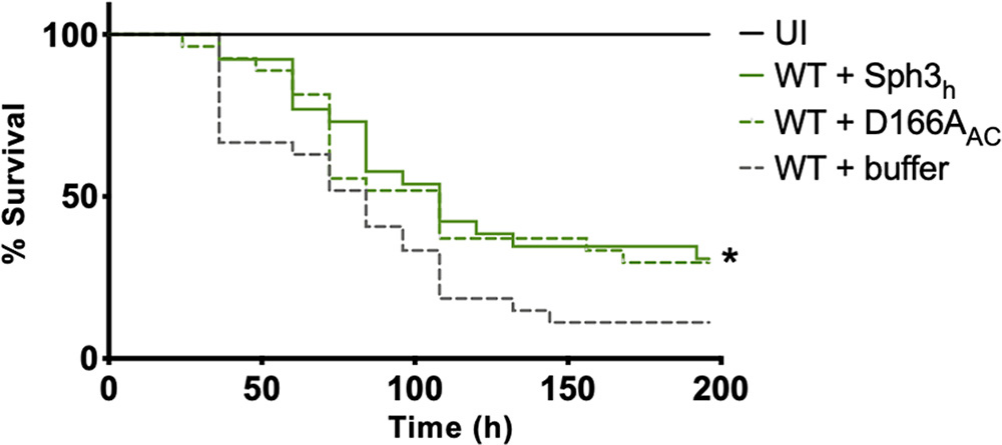
Catalytic activity of Sph3_h_ is dispensable for antifungal activity in a neutropenic mouse model of pulmonary invasive aspergillosis. Shown is survival of neutropenic mice that were intratracheally infected with 5 × 10^6^ wild-type (WT) A. fumigatus conidia coadministered with a single dose of 500 μg of Sph3_h_ or catalytically inactive Sph3_h_ variant (D166A_Ac_). Kaplan-Meier curves represent 3 independent experiments with ≥4 mice per group. A significant difference in survival between both Sph3_h_- and D166A_Ac_-treated groups compared with the uninfected (UI) group is indicated (*, *P < *0.0065). No significant difference was found between the wild-type-infected Sph3_h_-treated (WT + Sph3_h_) and the wild-type-infected D166A_Ac_-treated (WT + D166A_Ac_) groups as determined by Wilcoxon rank test (*P = *0.6975).

To confirm that the degradation of GAG was not required for the antifungal effects of GH prophylaxis, the antifungal activity of a catalytically inactive Sph3_h_ variant, D166A_AC_, was evaluated in A. fumigatus-infected neutropenic mice (Fig. 4A). Similar levels of protection were observed in A. fumigatus-infected mice receiving prophylaxis with wild-type Sph3_h_ and variant D166A_AC_ (Fig. 8). Collectively, these data suggest that Sph3_h_-antifungal activity is not mediated through enzymatic degradation of GAG.

## DISCUSSION

In this study, pulmonary administration of the GHs Sph3_h_, PelA_h_, or Ega3_h_-HEK was demonstrated to be well tolerated and induced minimal immune response in uninfected mice. Although Sph3_h_, PelA_h_, and Ega3_h_-HEK exhibited relatively short half-lives *in vivo*, intratracheal Sph3_h_ or Ega3_h_-HEK limited A. fumigatus growth in two *in vivo* models of invasive pulmonary aspergillosis, and all three agents prolonged survival of infected mice. Further, Sph3_h_ potentiated the activity of a commonly used antifungal, posaconazole, *in vivo* against invasive pulmonary aspergillosis. The work in this study demonstrates that GHs may have potential in the prevention of invasive aspergillosis.

Single-dose GH therapy was well tolerated and resulted in minimal changes in the pulmonary inflammatory response in the absence of infection. However, the GHs exhibited short half-lives, and the effects of repeated GH administration remain to be evaluated. Repeated GH dosing has been previously reported with a recombinant form of another P. aeruginosa GH with biofilm-disrupting activity, PslG_h_, and was found to be well tolerated in a chronic P. aeruginosa infection wound model (22). While these results are promising, more detailed immunotoxicity studies of multiple GH dosing and anti-GH antibody response are required to advance these agents toward use in clinical trials.

Individual GH enzymes exhibited differences in efficacy and half-life. Although intratracheal Sph3_h_, Ega3_h_-HEK, and PelA_h_ all enhanced survival after fungal challenge, only prophylaxis with Sph3_h_ and Ega3_h_-HEK limited A. fumigatus growth *in vivo*, while a trend toward reduced fungal burden was observed with PelA_h_. This observation is unlikely to be driven by more rapid PelA_h_ degradation, given that PelA_h_ exhibited a slightly longer half-life than Sph3_h_. Although this observation may simply reflect normal biological variability, it is possible that PelA_h_ is less active than the other GH enzymes. Consistent with this hypothesis, previous studies of A. fumigatus biofilm disruption *in vitro* reported that Sph3_h_ and Ega3_h_-*Pp* exhibited lower half-maximal effective concentrations than PelA_h_ (0.45 nM and 0.85 nM versus 2.80 nM, respectively) (12, 14).

The mechanism whereby GH enzyme therapy mediates improved survival and augments antifungal activity in invasive aspergillosis remains to be elucidated. Pharmacokinetics revealed that GHs have short half-lives *in vivo* relative to the growth rate of A. fumigatus (23), suggesting that there is a limited window where GAG and active GH enzymes are both present. Indeed, the efficacy of prophylaxis with the catalytically inactive Sph3_h_ variant and the early recruitment of neutrophils after Sph3_h_ therapy suggest that augmentation of innate pulmonary inflammatory responses underlie the antifungal activity of these agents. However, an important caveat to these studies is the fact that catalytically inactive GH enzymes can function as lectins that retain their ability to bind to their cognate polymers and can interfere with polymer function *in vitro* (17). It therefore remains possible that GH enzymes do mediate some of their effects *in vivo* via lectin-like interactions with GAG. GAG has been reported to mediate a wide variety of immunosuppressive and other effects *in vivo*, including cloaking cell wall glycans from host pattern recognition receptors, augmenting antifungal resistance, resisting damage by neutrophil extracellular traps, mediating hyphal adhesion, inducing neutrophil apoptosis, and inducing immunosuppressive IL-1 receptor antagonist secretion (5, 8–10). The relative contributions to virulence of each of these GAG-related phenotypes has not been well defined. These effects may become more apparent in investigations evaluating the mechanisms of GH efficacy with A. fumigatus isolates of various levels of virulence (24, 25) or with mouse strains with differences in innate or acquired immunity (26–28). BALB/c mice used in this study are intrinsically polarized toward a type two helper T-cell response following intravenous administration of A. fumigatus conidia (26). The use of additional strains of mice with differential helper T-cell protective responses against A. fumigatus could provide insight into the mechanism of action of the GHs. Further studies are required to probe the relative contribution of pulmonary inflammatory responses and other anti-GAG effects of GH enzymes on these mechanisms of fungal pathogenesis *in vivo.*

An important concern with biofilm-targeting therapies is the potential for dispersion of organisms following degradation of the extracellular matrix and a worsening of infection. This phenomenon has been reported *in vivo* with manipulation of P. aeruginosa biofilm regulatory pathways (29) as well as enzymatic treatment of P. aeruginosa biofilms with α-amylase and cellulase (30). In this study, GH therapy was associated with improved outcomes in A. fumigatus infection, and no clinical evidence of dissemination to the central nervous system was observed, although necropsy was not performed. These findings may reflect the early immune action of GH enzymes prior to the production of significant amounts of GAG but could also reflect fundamental differences in morphology and motility between molds and bacteria. In contrast to unicellular bacteria, molds grow as long multicellular filamentous hyphae that are intertwined and, thus, are less likely to be able to passively detach and disseminate (31). Further, unlike P. aeruginosa and many other bacteria, A. fumigatus hyphae are not motile and therefore are unable to actively disseminate from the site of infection (32–35). Consistent with the latter hypothesis, α-amylase- and cellulase-mediated dispersal of biofilms formed by a nonmotile bacterial species, Staphylococcus aureus, did not result in systemic bacterial dissemination in a chronic wound model (30). Together, these data suggest that morphology and the capacity for motility are important determinants that may partly drive the outcomes of biofilm-directed therapies.

For these initial studies of GH efficacy *in vivo*, a model of invasive aspergillosis in which GAG-covered hyphae invade and form fungal biomass around and within the lung parenchyma was used (4). One important future direction will be to investigate GH efficacy against fungal biofilms in a chronic pulmonary aspergillosis model. Concerns remain, however, about the use of the agar bead A. fumigatus chronic airway infection model (36), as the presence of a foreign extracellular matrix may compromise GH activity and penetration of GHs to the hyphal surface.

The results of this study demonstrate that intratracheal administration of Sph3_h_, PelA_h_, or Ega3_h_-HEK can improve survival during experimental invasive aspergillosis. The results of these studies lay the foundation for future work to elucidate the mechanism by which the GHs limit fungal growth *in vivo*, to test the efficacy of GHs in established infection, perform detailed immunotoxicity studies, and extend studies into a chronic pulmonary aspergillosis model. Further, nebulizable formulations for aerosolized GH deposition into the airways will need to be explored for eventual clinical use.

## MATERIALS AND METHODS

### Strain and growth conditions.

A. fumigatus strain Af293 (a generous gift from Paul T. Magee, University of Minnesota, St. Paul, MN) and the Δ*uge3* A. fumigatus strain (5) were grown on yeast extract-peptone-dextrose (BD Biosciences Difco) agar (BD Biosciences Difco) plates at 37°C, from −80°C stocks. Conidia were harvested following 6 days of growth with phosphate-buffered saline (PBS; HyClone) containing 0.1% (vol/vol) Tween 80 (PBS-T; Fisher Scientific), washed, and resuspended in PBS-T at either 1 × 10^5^ conidia/ml or 1 × 10^8^ conidia/ml for intratracheal infections.

### Recombinant GH expression and purification.

His-tagged PelA_h_, Sph3_h_, and catalytically inactive Sph3_h_ variant D166A_AC_ were expressed in Clearcoli cells grown in Terrific broth (Bioshop) with 50 μg/ml kanamycin (Biobasic) as previously described (11, 17, 37). Bacterial cultures were induced with 0.5 mM isopropyl-β-d-thiogalactopyranoside (IPTG) (Biobasic) when the cells reached an optical density at 600 nm (OD_600_) of 1.2 to 1.4. The cells were incubated postinduction overnight at 18°C with shaking at 200 rpm before being harvested by centrifugation at 5,000 × *g* for 30 min at 4°C. Both proteins were purified using Ni-nitrilotriacetic acid columns (GE Healthcare) followed by buffer exchange as previously described (37). The protein yield using this method was an average of approximately 80 to 100 mg/liter of culture.

Expression of Ega3_h_-*Pp* in the PichiaPink system was optimized as previously described (12). To generate a glycosylated Ega3_h_ that would mimic mammalian-like glycosylation patterns (38), Ega3_h_-HEK was expressed in a cell line of human embryonic kidney cells (HEK293). The region of the *ega3*^68–318^ gene was cloned using primers *ega3*-fwd (5′-GGGACCGGTGGTAATTATACCACCGCAAAATGG) and *ega3*-rev (5′-GGGGGTACCGCAATATTCCACCCA) from a pET28a vector (12) into a pHLsec vector under the control of a mammalian promoter. The plasmids were then transiently transfected into Freestyle 293 S (HEK293-S) cell lines for expression trials using FectoPro transfection reagent (VWR). The culture supernatants containing the secreted proteins were harvested at 3 and 6 days to measure protein yield. Six days was established as the essential incubation time for maximum protein expression. The cells were spun down, and the secreted His-tagged protein was purified from the supernatant by affinity chromatography followed by gel filtration using a HiLoad 16/600 Superdex 200 prep-grade column (GE Healthcare).

### Mice.

Six- to 8-week-old BALB/c female mice (Charles River Laboratories Inc., Senneville, QC, Canada, and Kingston, NY) were used for animal studies. Mice were anaesthetized with 4% isoflurane prior to intratracheal infection or prophylaxis/treatment with GHs/drug. Mice were monitored daily for signs of stress (ruffled fur, inactivity, and hunched posture), and body weights and temperatures were taken. Body weight was measured using a top-loading balance, and surface body temperature was taken on the abdomen using a digital infrared thermometer. Moribund animals were euthanized by isoflurane and CO_2_ overdose.

### Tolerability studies.

Immunocompetent mice were treated intratracheally with a single dose of Sph3_h_, PelA_h_, or Ega3_h_-*Pp* at 1, 5, 10, 100, or 500 μg in 50 μl PBS or PBS alone. Mice were monitored daily for 7 days for signs of illness, and body weights and temperatures were recorded. For histopathology studies, lungs from immunocompetent mice were inflated with 10% buffered formalin (Fisher Scientific) and fixed in formalin as previously described (14). Lungs were then embedded in paraffin, and 4-μm-thick sections were stained with hematoxylin and eosin. Scanned sections (Leica, Aperio) were analyzed with QuPath 0.1.2 software (39).

### Densitometry and antibody production.

SDS-PAGE and Western blotting techniques were used to assess pulmonary GH pharmacokinetics. Rabbit polyclonal antibodies specific to each of the GHs were produced by Cedarlane (Burlington, Canada) as previously described (37). Mice were treated intratracheally with a single dose of 500 μg of each GH and then euthanized, and their lungs were harvested at the indicated time points. Lungs were homogenized in a cocktail of protease inhibitors (Roche), and pulmonary GH concentrations were quantified by Western blotting with rabbit anti-GH antibodies. Goat-anti-rabbit horseradish peroxidase-conjugated secondary antibody (Bio-Rad) was detected with a chemiluminescent substrate (Thermo-Fisher). The half-life of each GH was determined by densitometric analysis using ImageJ software. Band intensity at each time point was normalized to the intensity at the zero-hour time point. Half-life was determined as 50% of the relative intensity of the bands compared to the zero-hour time point.

### Pulmonary damage.

Mice were treated intratracheally with a single dose of Sph3_h_, PelA_h_, or Ega3_h_-*Pp* at 100 or 500 μg in 50 μl PBS or PBS alone. Seven days after treatment, mice were euthanized and their lungs lavaged twice with 1 ml PBS as previously described (14). Lactate dehydrogenase activity was measured in the pooled bronchoalveolar lavage fluid with a commercial assay (CytoTox 96 nonradioactive cytotoxicity assay; Promega) per the manufacturer’s instructions.

### Pulmonary leukocyte quantification.

Immunocompetent mice were treated intratracheally with a single dose of Sph3_h_, PelA_h_, Ega3_h_-*Pp*, or Ega3_h_-HEK at 100 or 500 μg in 50 μl PBS or PBS alone. Seven days after treatment, mice were euthanized and their lungs were washed in PBS, minced in RPMI medium 1640 (Wisent) containing 5% (vol/vol) fetal bovine serum (FBS; Wisent), and then digested with 150 U/ml collagenase (Sigma) (14, 36). The resulting suspension was passed through a 70-μm cell strainer and treated with ACK buffer (Gibco). Approximately 1 × 10^6^ leukocytes were resuspended in a fixable viability dye (eBioscience) and washed, and their Fc receptors were blocked by unlabeled anti-CD16/32 antibodies (FcBlock; BD Pharmingen) as previously described (14). Cell surface components were then stained with fluorescently labeled antibodies (BD Biosciences) as previously described (14). Leukocytes were washed, fixed with paraformaldehyde (Electron Microscopy Sciences, Hatfield, PA), and then resuspended in PBS as previously described (14). Data were acquired on an LSR Fortessa flow cytometer with FACSDiva software (BD Biosciences) and analyzed with FlowJo software version 10 (FlowJo, LLC). Immune cell subsets were defined as previously described (14). Total cell populations were calculated by using the CountBright absolute counting beads (Invitrogen).

### Mouse models of invasive pulmonary aspergillosis. (i) Leukopenic mouse model.

Mice were rendered leukopenic by cortisone acetate (Sigma) and cyclophosphamide (Baxter) as previously described (6). Mice were intratracheally infected with a 50-μl suspension of 5 × 10^3^ A. fumigatus conidia in PBS-T as previously described (14).

### (ii) Neutropenic mouse model.

Mice were rendered neutropenic by intraperitoneal injection with anti-Ly6G antibody (clone 1A8; Bio X Cell) at 200 μg, starting 1 day prior to infection, every 48 h as previously described (18). Neutrophil depletion was confirmed by blood smear and differential staining. Mice were intratracheally infected with a 50-μl suspension of 5 × 10^6^ A. fumigatus conidia in PBS-T.

### Effects of GH prophylaxis in leukopenic and neutropenic mouse models of invasive pulmonary aspergillosis.

Leukopenic mice were intratracheally infected with a 50-μl suspension of 5 × 10^3^ A. fumigatus conidia in PBS-T and concomitantly treated with either a single dose of Sph3_h_, PelA_h_, or Ega3_h_-HEK at 500 μg in 50 μl of the corresponding buffer or buffer alone. At 1 and 2 or 4 days after prophylaxis, mice were euthanized and their lungs harvested for pulmonary leukocyte quantification as described above or pulmonary fungal burden determination as described below. Neutropenic mice were intratracheally infected and concomitantly treated with Sph3_h_, PelA_h_, Ega3_h_-HEK, or catalytically inactive Sph3_h_ variant D166_AC_ as described above and then monitored daily and euthanized upon reaching clinical endpoints.

### Sph3_h_-posaconazole combination prophylaxis in a neutropenic mouse model of invasive pulmonary aspergillosis.

Neutropenic mice were intratracheally infected with a suspension of 5 × 10^3^ A. fumigatus conidia and concomitantly treated with either a single dose of Sph3_h_ at 500 μg or buffer alone as described above, and beginning 12 h after infection they were treated by oral gavage with 2.5 mg/kg of body weight posaconazole or buffer alone every 12 h. Two days after the initiation of prophylaxis, mice were euthanized and their lungs were harvested for fungal burden determination as described below.

### Pulmonary fungal burden.

Lungs were harvested and homogenized in 5 ml PBS with a Polytron tissue homogenizer, and homogenates were stored at −80°C, modified from what was previously done (5). Pulmonary galactomannan content was determined by using the Platelia Aspergillus immunoassay kit (Bio-Rad) according to the manufacturer’s instructions as previously described (5, 14). The galactomannan values were then normalized to a highly infected lung homogenate standard.

### Statistical analysis.

Data are presented and statistical significance calculated as indicated. All graphs were generated and statistical analyses were performed in GraphPad Prism version 9.0.0 software. Significant differences between values were compared by two-way analysis of variance (ANOVA) with Dunnett’s multiple-comparison test, two-way ANOVA with Tukey’s multiple-comparison test, Kruskal-Wallis test with Dunn’s multiple-comparison test, or Wilcoxon rank test.

### Ethics statement.

All procedures involving mice were approved by the Animal Care Committees of the Institutional Animal Care and Use Committee (IACUC) of the McGill University Health Centre (protocol number 2016–7808) and Animal Care and Use Review Office (ACURO) of the United States Army Medical Research and Materiel Command (USAMRMC).

### Data availability.

We declare that the data supporting the findings of this study are available within the paper and its supplemental material.
