# Supplementary material for: Preclinical Evaluation of Recombinant Microbial Glycoside Hydrolases in the Prevention of Experimental Invasive Aspergillosis
Source: mBio. 2021 Sep 28;12(5):e02446-21. doi: 10.1128/mBio.02446-21 (PMC8546845; doi:10.1128/mBio.02446-21)
Supplement: FIG S1 [file mbio.02446-21-sf001.pdf]

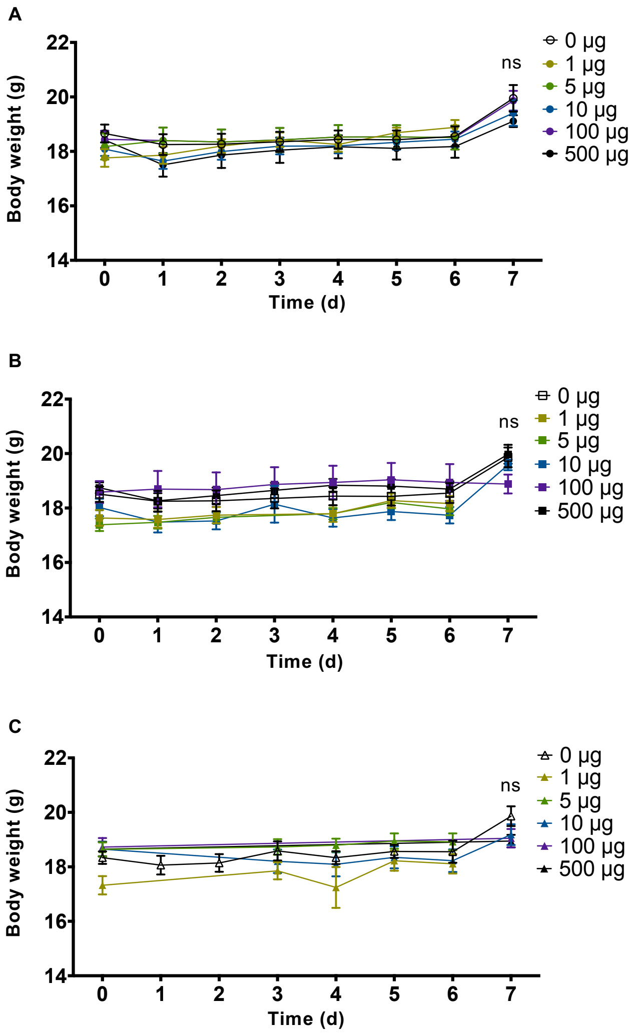

FIG S1. Intratracheal GH therapy is well tolerated by mice. Body weight of immunocompetent BALB/c mice following intratracheal treatment with the indicated single doses of (A) $Sph3_h$, (B) $PelA_h$, or (C) $Ega3_h$-*Pp*. Each point represents the body weight of ≥ 5 mice per group. ns indicates no significant difference in the change in body weight of mice treated with 500 µg GH relative to buffer-treated mice on the same day as determined by two-way ANOVA with Dunnett's multiple comparisons test.
