# Supplementary material for: Preclinical Evaluation of Recombinant Microbial Glycoside Hydrolases in the Prevention of Experimental Invasive Aspergillosis
Source: mBio. 2021 Sep 28;12(5):e02446-21. doi: 10.1128/mBio.02446-21 (PMC8546845; doi:10.1128/mBio.02446-21)
Supplement: FIG S3 [file mbio.02446-21-sf003.pdf]

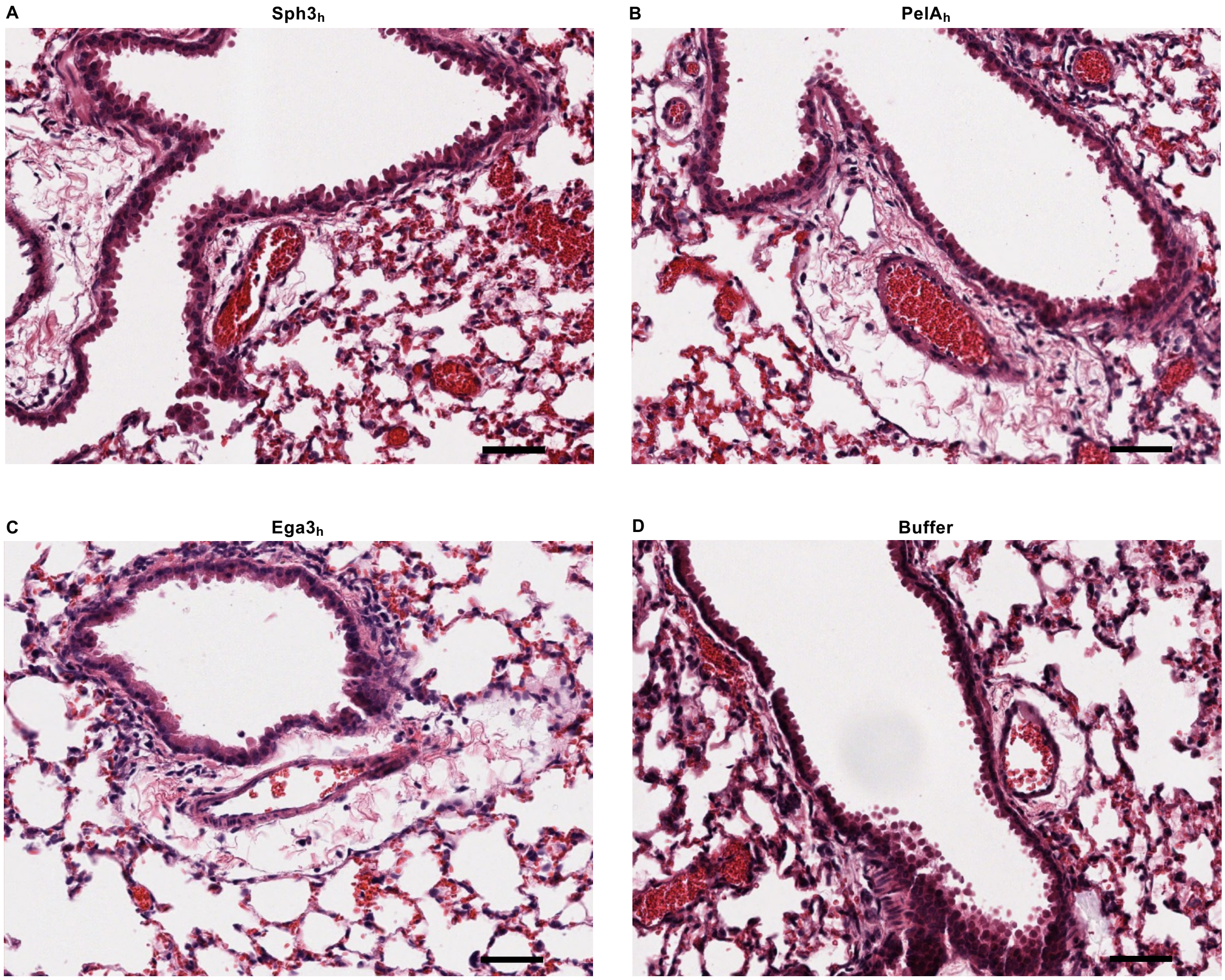

FIG S3. Intratracheal GH therapy is well tolerated by mice. Haematoxylin and eosin stained sections of lungs obtained from immunocompetent BALB/c mice 7 d after intratracheal treatment with a single dose of 500 µg of (A) $Sph3_h$, (B) $PelA_h$, (C) $Ega3_h$-*Pp* or (D) PBS. Representative images from 3 mice imaged at 40X (scale bar = 50 µm).
