# Supplementary material for: Preclinical Evaluation of Recombinant Microbial Glycoside Hydrolases in the Prevention of Experimental Invasive Aspergillosis
Source: mBio. 2021 Sep 28;12(5):e02446-21. doi: 10.1128/mBio.02446-21 (PMC8546845; doi:10.1128/mBio.02446-21)
Supplement: FIG S4 [file mbio.02446-21-sf004.pdf]

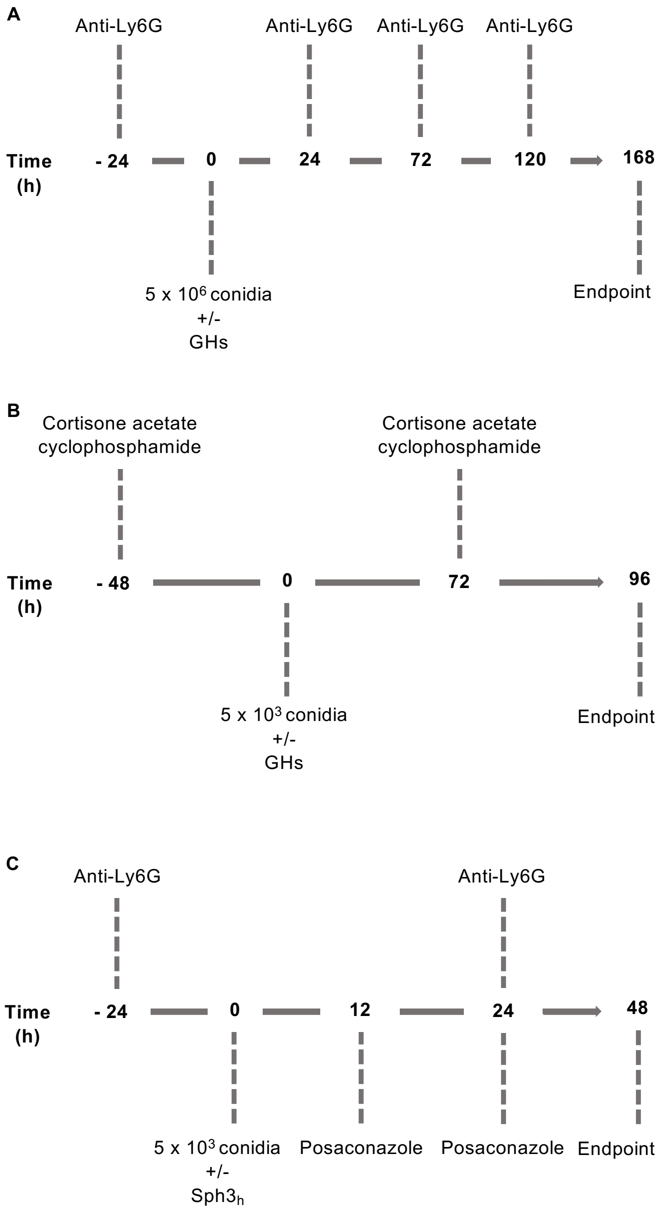

FIG S4. Experimental design of GH evaluation in immunocompromised mouse models of invasive aspergillosis. (A) Neutrophil depletion model: mice were rendered neutropenic by intraperitoneal injection of 200 µg anti-Ly6G antibody starting 24 h prior to infection, and every 48 h thereafter. Mice were then intratracheally infected with 5 x 10^6 *A. fumigatus* conidia with, or without GHs and survival was monitored. (B) Leukopenia model: mice were rendered leukopenic by subcutaneous injection with 250 mg of cortisone acetate per kg of body weight 48 h prior to infection and at 72 h after infection; and by intraperitoneal injection with 250 mg cyclophosphamide per kg 48 h prior to infection and 200 mg at 72 h after infection. Mice were intratracheally infected with 5 x 10^3 *A. fumigatus* conidia with and without GHs and pulmonary fungal burden was measured at 96 h. (C) $Sph3_h$-posaconazole combination studies: mice were rendered neutropenic as in a.). Mice were intratracheally infected with 5 x 10^3 *A. fumigatus* conidia co-administered with or without single dose of 500 µg $Sph3_h$ then treated by oral gavage with 2.5 mg/kg posaconazole every 12 h and pulmonary fungal burden was measured 48 h after infection.
